# Ultra-Stretchable Piezoelectric Nanogenerators via Large-Scale Aligned Fractal Inspired Micro/Nanofibers

**DOI:** 10.3390/polym9120714

**Published:** 2017-12-15

**Authors:** Yongqing Duan, Yajiang Ding, Jing Bian, Zhoulong Xu, Zhouping Yin, Yongan Huang

**Affiliations:** 1 State Key Laboratory of Digital Manufacturing Equipment and Technology, Huazhong University of Science and Technology, Wuhan 430074, China; duanyongqing@hust.edu.cn (Yo.D.); dyjqwert@gmail.com (Ya.D.); jsbianjing@hust.edu.cn (J.B.); xuzhoulong@hust.edu.cn (Z.X.); yinzhp@hust.edu.cn (Z.Y.); 2 Flexible Electronics Research Center, Huazhong University of Science and Technology, Wuhan 430074, China

**Keywords:** stretchable electronics, nanogenerators, piezoelectric nanofibers, electrospinning, buckling

## Abstract

Stretchable nanogenerators that directly generate electricity are promising for a wide range of applications in wearable electronics. However, the stretchability of the devices has been a long-standing challenge. Here we present a newly-designed ultra-stretchable nanogenerator based on fractal-inspired piezoelectric nanofibers and liquid metal electrodes that can withstand strain as large as 200%. The large-scale fractal poly(vinylidene fluoride) (PVDF) micro/nanofibers are fabricated by combination of helix electrohydrodynamic printing (HE-Printing) and buckling-driven self-assembly. HE-Printing exploits “whipping/buckling” instability of electrospinning to deposit serpentine fibers with diverse geometries in a programmable, accurately positioned, and individually-controlled manner. Self-organized buckling utilizes the driven force from the prestrained elastomer to assemble serpentine fibers into ultra-stretchable fractal inspired architecture. The nanogenerator with embedded fractal PVDF fibers and liquid-metal microelectrodes demonstrates high stretchability (>200%) and electricity (currents >200 nA), it can harvest energy from all directions by arbitrary mechanical motion, and the rectified output has been applied to charge the commercial capacitor and drive LEDs, which enables wearable electronics applications in sensing and energy harvesting.

## 1. Introduction

Piezoelectric nanogenerators (NGs) that directly convert mechanical energy into electrical energy have drawn tremendous attention because of their great potential for mechanical energy harvesting, sensing, and actuation. In addition to high output performance, integration of flexibility and stretchability to NGs has great research significance that enables applications in flexible/stretchable electronic systems, such as epidermal electronics, e-skin, and soft bionic devices [1,2,3]. Particularly in the case of epidermal electronics, soft and curved human body is covered with highly extensible skins with average strain from 3 to 55% [4,5], therefore, the NGs should accommodate strain up to >100% for the attachment and detachment on/from skin in a safe manner. In the past decades, tremendous efforts have been committed to the design and fabrication of conformal piezoelectric energy harvesters for power generation from the natural motions of the heart, lung, and diaphragm, and the electrical performance of the NGs has been significantly improved [6,7]. However, a large number of flexible NGs only work at very low strain levels because the functional materials are of very low fracture strain thresholds (≈1% for zinc oxide (ZnO), ≈0.2% for lead zirconate titanate (PZT), ≈2% for poly(vinylidene fluoride) (PVDF)) [2]. To enhance stretchability, the piezoelectric materials have been designed into mechanical stretchable architectures, e.g., buckled/serpentine/fractal structures and textiles [8,9,10,11,12]. Qi et al. fashioned the PZT ribbons into a wavy-shaped pattern, the out-of-surface buckled PZT ribbons integrated with stretchable PDMS rubber can be stretched up to 8% strain [8]. The current mainstream strategy in fabrication still depends on complicated and expensive lithography process and transfer printing, instead of a cost-effective printing process. Meanwhile, the ribbon-based structures inevitably form out-of-surface deformation rather than in-surface deformation [13,14,15], which easily causes irreversible deformation and increases the encapsulation difficulty.

Compared to nanoribbons, nanofiber-based generators are expected to be more flexible, maneuverable, and conformable. Gu et al. developed a high-output nanogenerator based on vertically-aligned, ultralong PZT nanofibers fabricated using electrospinning technology. The device was capable of being bent, stretched, or twisted to a large degree without breaking [16]. Zhong et al. prepared a fiber-based generator for wearable electronics, the fiber-based generator was identified as an effective building element in textiles for smart garments with stretchability of 2.15% [17]. Electrospun fibers of the PVDF polymer are an important candidate for flexible/stretchable nanogenerator applications due to their particularly attractive piezoelectric properties, structural flexibility, biocompatibility, and ease of processing [18,19,20], however, most of them are in the form of non-woven mats or straight micro/nanofibers [21,22,23,24,25] with limited flexibility. It is of great significance to design a fiber-based generator with stretchability much larger than 100% and stable electrical output for developing wearable electronics and practical self-powered electronic devices.

Here we report an ultra-stretchable nanogenerator realized by fractal serpentine PVDF nanofibers and liquid metal microelectrodes, where the former is fabricated through the helix electrohydrodynamic printing (HE-Printing) technique and self-organized buckling. HE-Printing exploits the “whipping/buckling” phenomenon of the electrospinning jet to directly fabricate serpentine fibers with diverse geometries in a programmable, accurately positioned, and individually-controlled manner and, in situ, electrically polarizes piezoelectric PVDF nanofibers. The serpentine micro/nanofibers transferred onto a prestrained elastomer buckle into fractal structures when releasing the prestrain of elastomer. The ultra-stretchable NGs with fractal PVDF fibers demonstrate high mechanical (stretchability > 200%) and electrical (currents up to 200 nA) performances. They can harvest energy from all directions by biomechanical motion via various stimuli, such as bending, stretching, twisting, etc., demonstrating their great potential in wearable electronics applications.

## 2. Experimental Section

### 2.1. Fabrication of Ultra-Stretchable NGs

**Figure 1:**
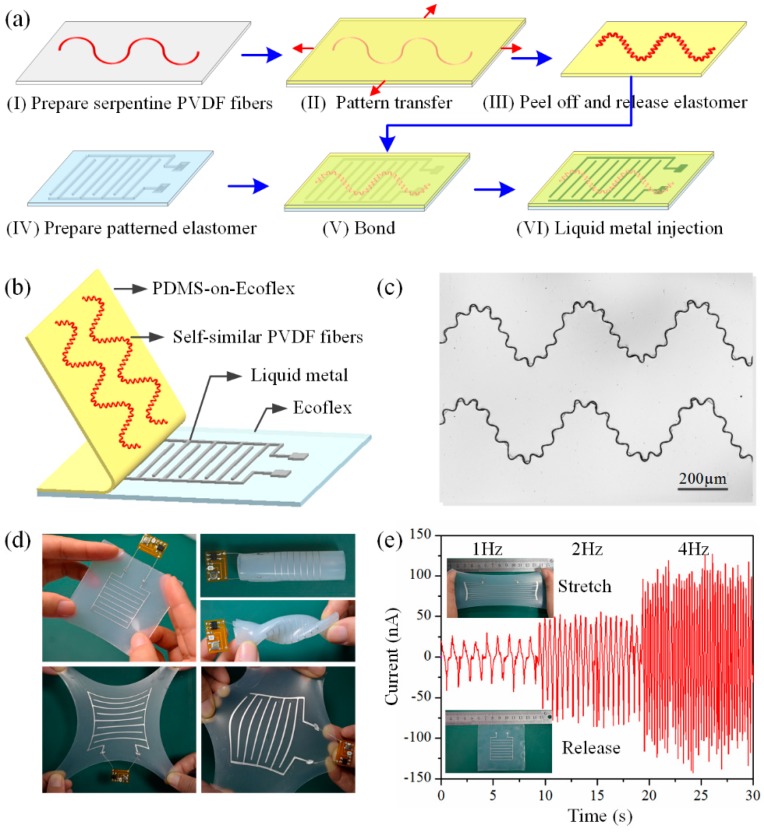
(**a**) Fabrication process of the stretchable nanogenerator; (**b**) Schematic diagram of the stretchable nanogenerator with fractal micro/nanofibers; (**c**) Optical image of fractal PVDF micro/nanofibers; (**d**) Photographs of the nanogenerator under various deformations, including bending, twisting, and uniaxial/biaxial stretching; (**e**) Output currents of the nanogenerator with different stretch and release frequencies under an applied strain of 200%.

The fabrication process for the ultra-stretchable NGs was shown in Figure 1a: (I) Prepare highly aligned serpentine PVDF micro/nanofibers by HE-Printing. The polymer PVDF (Kynar 761, Arkema, Colombes, France, 18 wt %) was added in the solvent mixture DMF/acetone (50/50 wt %), and heated at 35 °C for 6 h to make the solution homogeneous. The PVDF solution was delivered using a syringe pump and electrospun into ultrafine micro/nanofibers with diameters ranging from hundreds of nanometers to a few micrometers by exerting a high voltage between a stainless-steel needle (inner/outer diameter of 260 μm/510 μm) and an aluminum collector through a power supply (DW-P403, Dongwen, Inc., Tianjin, China). Three key process parameters, the applied voltage, the nozzle-to-collector distance, and the collector velocity, were adjusted to obtain electrospun fibers with various geometries; (II) Transfer the serpentine PVDF micro/nanofibers by placing the bidirectionally-prestrained PDMS-on-Ecoflex elastomer onto a silicon substrate on which PVDF micro/nanofibers have been deposited. PDMS-on-Ecoflex substrate was prepared by spin-coating PDMS solution (sylgard 184, Dow Corning, Inc., Midland, MI, USA) onto a cured Ecoflex substrate (Ecoflex 0030, Smooth-On, Inc., Easton, PA, USA), and thermally cured at 60 °C for 40 min to obtain a half-cured thin PDMS layer with a thickness of about 0.1 mm; (III) Peel off and release the prestrain of the PDMS-on-Ecoflex elastomer to obtain the buckling-driven fractal micro/nanofiber; (IV) Prepare an Ecoflex substrate with micro-channels by pouring the Ecoflex solution (Ecoflex 0030, Smooth-On Inc.) into a mold and cure at 60 °C for 10 min; (V) Bond the micro-patterned Ecoflex substrate with the PDMS-on-Ecoflex substrate to form micro-channels with a cross-section of 400 μm × 400 μm; (VI) Inject liquid metal (EgaIn, 75% gallium and 25% indium) into the micro-channels to achieve ultra-stretchable interdigital electrodes. The final device (with the size of about 6.5 cm × 6.5 cm) is shown in Figure 1d,e, and the number of PVDF fibers integrated in the device ranges from 20 to 150.

### 2.2. Characterization

A laser scanning confocal microscope (LSCM, Keyence VK-X200K, Osaka, Japan) and a scanning electron microscope (SEM, Sirion 200, FEI, Hillsboro, OR, USA) were employed to observe the fiber morphology. The Fourier transform infrared (FTIR) spectra were recorded on a Perkin-Elmer spectrum 100 equipped with an infrared grid polarizer (Specac Limited, Orpington, UK). A semiconductor parameter analyzer (Keithley 4200-SCS, Cleveland, OH, USA) and a probe system (Cascade Summit 11000, FormFactor Livermore, CA, USA) were utilized to characterize the piezoelectric properties of NGs, where the device was fixed on a home-made tensile platform whose tensile speed, amplitude and frequency can be tuned digitally, or attached to human bodies with tape to monitor or harvest energy from human movement.

## 3. Results

Figure 1b schematically illustrates the ultra-stretchable, fractal serpentine fiber-based nanogenerator, the sandwich-structured composite consists of four layers, a layer with fractal PVDF micro/nanofibers for electricity generation, a liquid metal layer for charge transfer, and two elastomer layers for encapsulation. All these four layers are designed with ultra-stretchability to improve the mechanical performance of the integrated device. The fabrication process is shown in Figure 1a. First is to direct-write serpentine micro/nanofibers on a silicon substrate through HE-Printing, then the serpentine fibers are transferred onto a bidirectionally-stretched PDMS-on-Ecoflex substrate to form self-organized fractal structures. PDMS-on-Ecoflex substrate is utilized here considering the surface stickiness of fresh PDMS to achieve a high bonding strength with serpentine micro/nanofibers, and the ultra-elasticity of Ecoflex to improve device stretchability. Figure 1c shows the optical image of the fractal serpentine fiber array, there have been dozens of small waves in one cycle of first-level serpentine fibers, and the spring within spring structure is promising for achieving ultra stretchability. The fractal fibers on elastomer are bonded to a patterned Ecoflex substrate to form microchannels, followed by injecting liquid metal acting as ultra-stretchable interdigital electrodes. Figure 1d shows the photographs of the NGs under various deformations, e.g., bending, twisting and stretching. It exhibits stable electrical output under an applied strain of 200% at different stretch and release frequencies (Figure 1e). Furthermore, the ultimate tensile capacity of the device can reach 300% as shown in Supplementary Figure S1, which demonstrates the ultra stretchability of the fractal fiber-based NGs.

**Figure 2:**
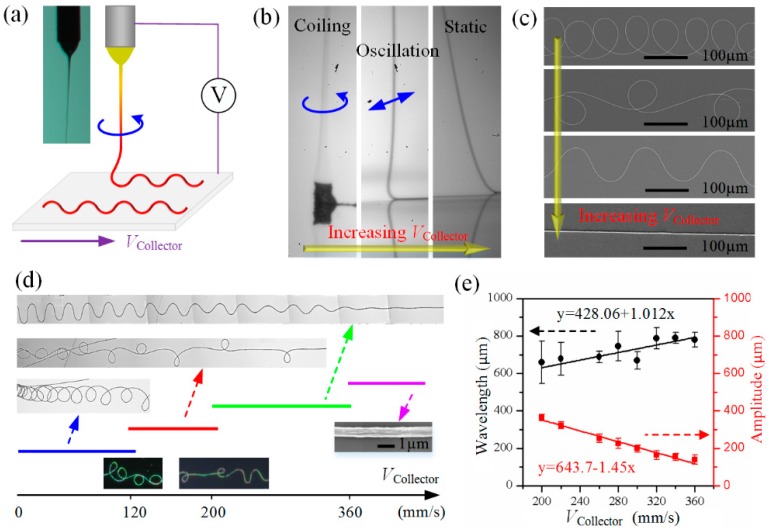
(**a**) The schematic diagram of HE-Printing; (**b**) Optical images of the electrospun jet under different collector velocities; (**c**) SEM images of various patterns direct-written on collector under different collector velocities; (**d**) Patterns deposited on collector when continuously increasing the collector velocity; (**e**) Wavelength/amplitude of serpentine fibers versus collector velocity.

To achieve spring within spring fractal structures, the first step is to realize controllable direct-writing of serpentine PVDF micro/nanofibers through HE-Printing, as shown in Figure 2a. Different from uncontrollable whipping of traditional electrospinning caused by electrical driven bending instability and a straight jet of near-field electrospinning [26], HE-Printing controls the fiber flying in a helical manner by introducing the rope coiling effect [27,28] into electrospinning process, and the flying micro/nanofiber consists of a long, straight “tail” that deforms by severe electrical stretching, and a short, helical “coil” that deforms under bending and twisting. Relatively large nozzle-to-collector distance (10–50 mm) was adopted in HE-Printing to realize tunable solidification of the electrospinning jet, meanwhile uncontrollable whipping should be prevented. The solidified electrospinning fiber buckles and rotates at impingement on a collector surface, which accumulates into a cylinder at the case of a stationary collector (left image of Figure 2b), and bifurcates into a series of complex patterns (Figure 2c) via the linear movement of collector. When the collector velocity is larger than the jet velocity, the lowermost part of the filament evolves from an oscillated buckled “heel” into a static stretched catenary (middle and right image of Figure 2b), and the patterns deposited on collector transition from serpentine to straight fiber (Figure 2c). The reproducible electrospun patterns are almost identical in the gravity driven rope coiling [29], although they have different characteristic scales.

**Figure 3:**
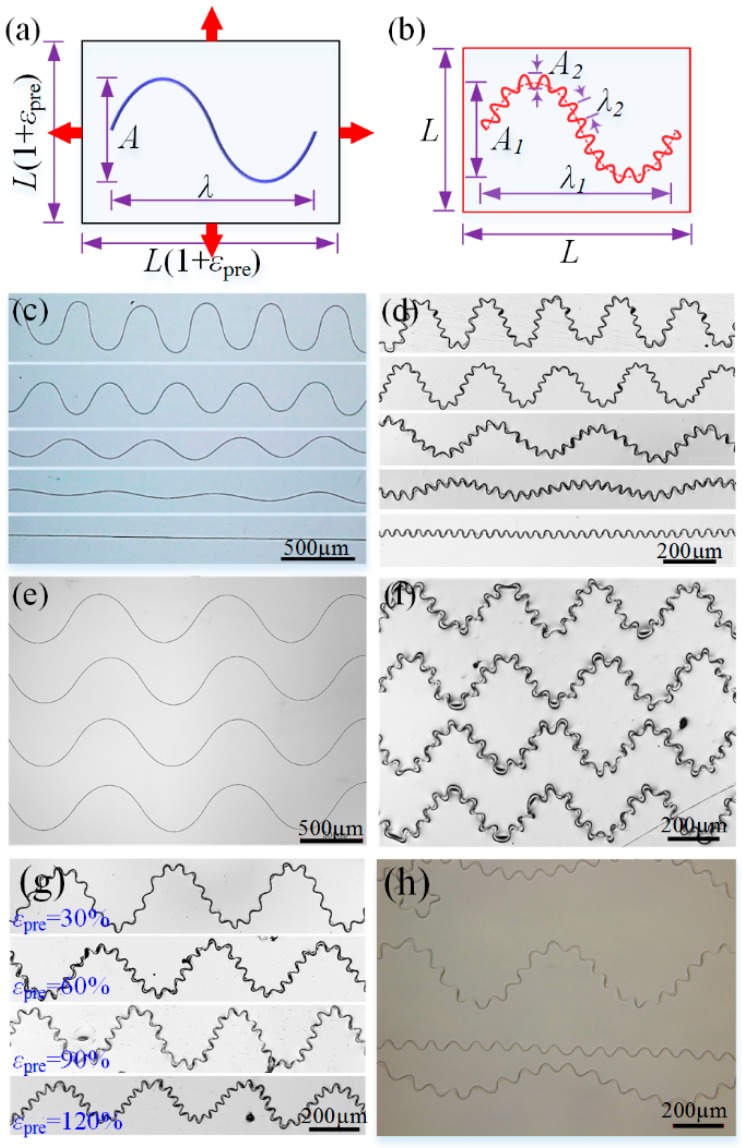
(**a**,**b**) Schematic diagram of serpentine micro/nanofibers on prestrained and prestrain-released elastomers; (**c**) Serpentine micro/nanofibers direct-written with different wavelength and amplitude; (**d**) Fractal micro/nanofibers fabricated basing on (**c**); (**e**,**f**) The large-area direct-written serpentine micro/nanofibers and fractal fiber-based structures; (**g**) Fractal micro/nanofibers fabricated under different prestrains; (**h**) Serpentine/straight micro/nanofibers generated under the same prestrain.

Collector velocity is the key factor that determines the geometry of the direct-written curve. Figure 2d exhibits the patterns when continuously increasing the collector velocity, where the applied voltage is 2.5 kV, the nozzle-to-collector distance is 25 mm. With the increase of collector velocity, translated coiling, alternating loops, serpentine fibers, and straight fibers appear in succession, and the critical speeds for pattern transformation is about 120, 200 and 360 mm/s, respectively. For each curve pattern, its structure gets looser when increasing collector velocity (the inset graphs in Figure 2d), and gradually convert to another shape during the pattern transition region. For serpentine fibers adopted here, its wavelength linearly increases with the collector velocity, while the amplitude shows a linear decreasing function ending with a straight fiber as shown in Figure 2e. Under subtle control of collector velocity, serpentine fiber-based microstructures with various wavelength/amplitude ratios can be fabricated as shown in Figure 3c.

In addition to collector velocity, the applied voltage and nozzle-to-collector distance also play an important role in determine pattern type and size. As shown in Figure 2a, a high voltage is exerted between nozzle and collector to generate the Taylor cone and pull out the jet from nozzle to collector. Higher voltage or smaller nozzle-to-collector distance correspond to stronger electrical filed, thus a larger falling speed and a smaller jet diameter can be achieved due to the more severe electrical stretching. Large falling speed indicates that large collector velocity is needed to stretch the jet from curve to straight (as shown in Figure 2d) [30,31,32]. Small jet diameter corresponds to small bending/torsional stiffness, thus small buckled patterns deposited on collector [27,28].

The fractal design of the fiber-based nanogenerator represents an important approach to simultaneously achieve excellent stretchability and tunable electrical generation. Ultra-stretchable fractal serpentine structures have been achieved by transferring the direct-written initial serpentine fibers onto a biaxial prestrained PDMS-on-Ecoflex substrate, then releasing the prestrain of elastomer. Figure 3a,b illustrates the generation principle of fractal geometry from the buckling of serpentine fiber. When releasing the biaxial prestrain of the elastomer, the initial serpentine geometry (*λ* and *A*) proportionally shrinks to the first-level wavy geometry (*λ*_1_ and *A*_1_), meanwhile buckling to the second-level wavy geometry (*λ*_2_ and *A*_2_) due to compressive strain. As demonstrated above, serpentine structures with specific ratios of amplitude and wavelength (Figure 3c) can be formed by digitally tuning the process parameter, e.g., the collector velocity, and these fibers are all able to form regular fractal structure (Figure 3d) when transfer them onto equal biaxial prestretched elastomer. Figure 3e,f show optical images of large-scale direct-written serpentine fiber array and self-assembled fractal fiber array. However, when the prestrains of the elastomer in two directions are not equal, the buckling behavior of the serpentine fiber becomes complicated, as shown in Supplementary Figure S2.

Figure 3g shows the buckling of serpentine fibers by prestrains from 30 to 120%, the prestrain decreases the size of the first-level serpentine structure, and increases the density of the second-level buckled geometry. The first-level wavelength and amplitude can be geometrically derived as *λ*_1_ = *λ*/(1 + *ε_pre_*) and *A*_1_ = *A*/(1 + *ε_pre_*), where *ε_pre_* is the biaxial prestrain of elastomer. The second-level wavelength and amplitude are difficult to directly calculate, but they are approximately equal to the buckling wave organized from straight fiber under the same prestrain (Figure 3h). For straight fibers, the initial buckling wavelength and amplitude can be derived as $\lambda_{2}=\frac{14\pi }{5}{\left(\frac{E_{fiber}I_{fiber}}{{\bar{E}}_{s}}\right)}^{\frac{1}{4}}$ and $A_{2}=\frac{\lambda_{2}}{\pi }\sqrt{\varepsilon_{pre}-\varepsilon_{critical}}$, where the critical strain for inducing buckling $\varepsilon_{critical}=\sqrt{\frac{{\bar{E}}_{s}}{E_{fiber}}}\frac{\sqrt{I_{fiber}}}{A_{fiber}}(\frac{1}{2}+\frac{2\pi }{5-2\gamma -2\ln(5/7)-0.5\ln(W^{4}{\bar{E}}_{S}/E_{fiber}/I_{fiber}/16)})$, ${\bar{E}}_{s}=E_{s}/\left(1-\nu_{s}^{2}\right)$, $E_{fiber}$, $I_{fiber}$, $A_{fiber}$ are the Young’s modulus, cross-sectional moment of inertia, and sectional area of fiber, $E_{s}$, $\nu_{s}$ are the Young’s modulus and Poisson’s ratio of the elastomer substrate, and *W* represents the contact width between fiber and substrate [33].

The buckling mode is also an important issue. Traditional fractal structures are mostly fabricated through lithography and etching, and the nanoribbon-based architecture inevitability experiences out-of-plane buckling or wrinkling under large stretching [11]. To ensure stretchability, freestanding encapsulation of this structure is needed, and it is difficult to implement. Our fiber-based fractal structure adhered to the elastomer maintains in-plane deformation under very large strain (Supplementary Figure S3) as the fiber cross-section is nearly circular, this reduces the difficulty of encapsulation and benefits the structural stability.

**Figure 4:**
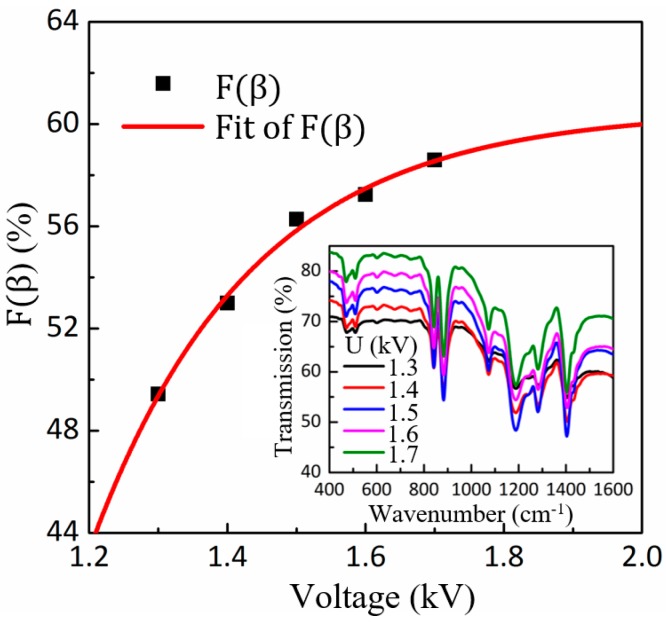
The concentration of β phase *F*(β) versus the applied voltage, the inset graph shows the FTIR transmission spectrum of the PVDF fibers.

Electrical polarization for piezoelectricity of PVDF is to transform the basic crystalline phases (α and γ) into the polar β phase, which is responsible for its piezoelectric properties. Figure 4 shows the FTIR transmission spectrum of the PVDF fibers electrospun with applied voltages ranging from 1.3 to 1.7 kV, the strong electric fields (greater than 10^6^ V/m) and stretching forces from the electrospinning process naturally align the dipoles in the nanofiber crystal such that the nonpolar phase (random orientation of dipoles) is transformed into the polar β phase, which indicates that in situ electrical poling in HE-Printing can polarize PVDF along the fiber without post-processing. In order to determine the fraction of the content of the β phase, infrared spectroscopy absorption bands at 795 and 840 cm^−1^ are chosen to characterize the α and β phases. The relative fraction of the β phase *F*(β) can be calculated by using *F*(β) = *X*_β_/(*X*_α_ + *X*_β_) = *A*_β_/[(*K*_β_/*K*_α_)*A*_α_ + *A*_β_] = *A*_β_/(1.26*A*_α_ + *A*_β_), where *X*_α_ and *X*_β_ are the crystallinity degree of α and β phases, *A*_α_ and *A*_β_ are the absorbance at 795 and 840 cm^−1^, and *K*_α_ = 6.1 × 10^4^ cm^2^/mol and *K*_β_ = 7.7 × 10^4^ cm^2^/mol are the absorption coefficients at the respective wave numbers [34]. Figure 4 indicates that the concentration of the β phase *F*(β) increases monotonously with the applied voltage, but converges to about 60%. For the electric poling effect, the cells of the α phase chain rotate to align their dipole moments in the direction favored by the external electric field, which increase the β phase content. In addition to the applied voltage, the tensile force from substrate is also capable of controlling the polarizability. Ding et al. have showed that *F*(β) increased linearly with the speed of the substrate within the range of 0 to 350 mm/s [35]. Both ends of the electrospun jet are constrained by the nozzle and substrate, so when increasing the substrate velocity, the mechanical drawing force applied to the fiber by the substrate increases, which results in more chains aligning, and the larger the β phase content.

**Figure 5:**
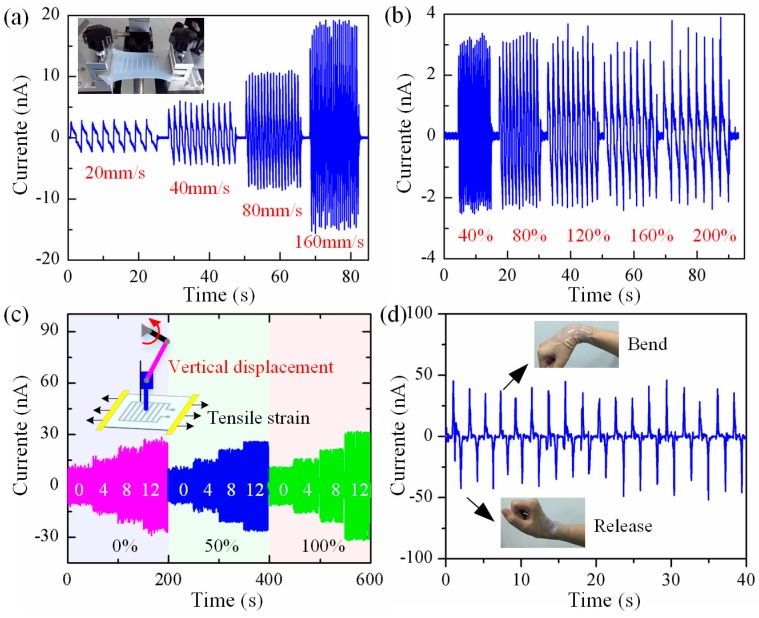
(**a**) The output current of 120% stretching in uniaxial direction under 20, 40, 80 and 120 mm/s; (**b**) The output current of 40, 80, 120, 160 and 200% stretching in the uniaxial direction, under the same velocity of 40 mm/s; (**c**) Current output of a freestanding nanogenerator subjected to various vertical displacement (0, 4, 8 and 12 mm) under different applied strains (0%, 50% and 100%); (**d**) The electrical output for monitoring human wrist movement.

Figure 5 shows the output performance of the nanogenerator under various deformations, it is capable of generating electricity from all directions by arbitrary mechanical motion in the environment. Figure 5a shows the electrical output of reciprocating stretching the device through a homemade tensile platform under an applied strain of 120%, the average maximum currents are 2.5, 5, 10, and 20 nA, corresponding to the stretching speeds of 20, 40, 80, and 160 mm/s, respectively. This indicates that the electrical outputs linearly increase with the tensile load speed. These results show that the performance characteristics of buckled piezoelectric PVDF fibers are consistent with fundamental piezoelectric theory $i=d_{33}EA\dot{\varepsilon }$ [36], where *i* is the output current, *d*_33_, *E*, and *A* are the piezoelectric charge constant, Young’s modulus, and cross-sectional area of PVDF fibers, and $\dot{\varepsilon }$ is the applied strain rate of PVDF fibers which is proportional to the tensile load speed. Figure 5b illustrates the outputs approximatively keep constant under the uniform motion of 40 mm/s, even the strain amplitude changes from 40 to 200%. And similar results can be obtained along biaxial stretching in Supplementary Figure S4. These results indicate that the electrical outputs of both uniaxial and biaxial stretching are determined by the speed of tensile load, rather than the amplitude. Figure 5c shows the output results of the freestanding nanogenerator under various vertical displacement loadings and tensile strains through a crank-slider mechanism. The device is first clamped through a home-made tensile platform, whose status can be originally clamped or be stretched. Then a vertical displacement is exerted on the device through a crank-slider mechanism, the loading displacement can be adjusted by the distance between the crank-slider mechanism and the tensile platform/device, as shown in the schematic diagram in Figure 5c. The output currents notably increase when varying the vertical displacement from 0 to 12 mm under the same tensile strain, and basically unchanged when stretching the device from 0 to 100% under the same vertical load, proving that the output current is mainly determined by the vertical displacement/pressure other than the applied tensile strain. Supplementary Figure S5a indicates that the device also has very small detection limit, it can detect the falling down of a paper as small as 0.2 mg. Figure 5d demonstrates that the stretchable nanogenerator has potential applications in monitoring human movement, it is capable of generating electricity with different wrist motions when the device was attached comfortably to the human wrist. The electrical output keeps stable after 1400 times of stretching 150% with frequency 1.25 Hz, demonstrating excellent durability of the device (Supplementary Figure S5b). These stable and sensitive characteristics of the nanogenerator are beneficial for artificial skin applications.

**Figure 6:**
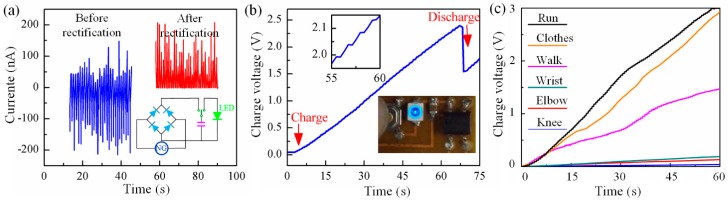
(**a**) Electrical output for energy harvester before and after rectification; (**b**) Voltage of a capacitor as a function of time during charging by fractal fibers under cyclic stretching load, and the charging and discharging by a LED lighter; (**c**) The charging rates of various body motions.

In many practical applications of wearable electronics, the ability of the nanogenerator to charge a capacitor is valuable. Figure 6a shows the current output of the nanogenerator when it is driven by stretching and releasing, as well as the rectified output and the schematic diagram of the rectification circuit. The rectified outputs from the stretchable energy harvester can be captured directly by using a chip-scale rechargeable battery (0805 chip capacitor with capacitance of 4.7 μF), an LED (3528 chip LED), and a Schottky bridge rectifier (MB12S; Micro Commercial Components, Simi Valley, CA, USA) integrated on a flexible substrate. Figure 6b shows the charging voltage of the capacitor, each ladder represents a cycle of stretching and releasing under an applied strain of 200% at a frequency of 1 Hz. As the process continues, the voltage of the capacitor increases and the collected energy is capable of lighting a commercial LED. Figure 6c shows charging curves of the capacitor when the energy harvester is mounted on various body parts with different motions, including wrist, elbow, knee, and the sole of the foot at walking and running. Furthermore, it can be stuck to clothes to collect energy when shaking the clothes. The concrete testing images are shown in Supplementary Figure S6, and each ladder in Figure 6c also represents a reciprocating cycle. The charging efficiency is very close for the energy harvester on the sole of foot at running and on the cloth at shaking, which are far higher than the other working status. About 20 fibers are integrated in the current nanogenerator configuration, and the efficiency of the charging system can be improved by increasing the number of piezoelectric fibers through HE-Printing, without a reduction of the stretchability.

## 4. Conclusions

The ultra-stretchable NGs reported here provide routes to energy harvesting systems that are attractive for powering highly stretchable electronic/optoelectronic devices. The nanogenerator composed of fractal fiber-based microstructures and embedded liquid-metal electrodes show high mechanical (stretchability up to 200%) and electrical (output currents up to 200 nA) performances under reciprocating deformation tests, with the ability to realize omnidirectional electricity generation. The HE-Printing technique for versatile and rapid fabrication of serpentine piezoelectric PVDF fibers in a controlled manner, and a fractal design that offers unique, spring within spring structure for enhanced stretchability, have been proposed. The essential features of process responsible for formation of such serpentine fibers have proved crucial to both scientific understanding and engineering application, and assembling of fractal fibers will serve as building blocks of stretchable electronics. Experimental and theoretical studies presented hereby provide guidelines for the design, fabrication, and operation of ultra-stretchable nanogenerators.

## Supplementary Materials

The supplementary materials are available online at www.mdpi.com/2073-4360/9/12/714/s1.
