# Supplementary material for: Ultra-Stretchable Piezoelectric Nanogenerators via Large-Scale Aligned Fractal Inspired Micro/Nanofibers
Source: Polymers (Basel). 2017 Dec 15;9(12):714. doi: 10.3390/polym9120714 (PMC6418612; doi:10.3390/polym9120714)
Supplement: Supplementary file 1 [file polymers-09-00714-s001.pdf]

Supplementary Information

Ultra-Stretchable Piezoelectric Nanogenerators via Large-Scale Aligned Fractal Inspired Micro/Nanofibers

Yongqing Duan[1,2], Yajiang Ding[1,2], Jing Bian[1,2], Zhoulong Xu[1,2], Zhouping Yin[1,2], YongAn Huang[1,2,*]

1. State Key Laboratory of Digital Manufacturing Equipment and Technology, Huazhong University of Science and Technology, Wuhan, 430074, China
2. Flexible Electronics Research Center, Huazhong University of Science and Technology, Wuhan, 430074, China

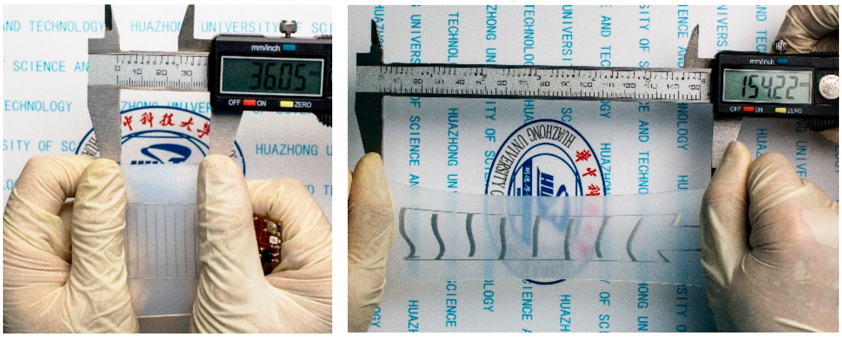

Figure S1. The photographs of the device before and after stretching, which demonstrate that the ultimate tensile capacity of the device can reach 300%.

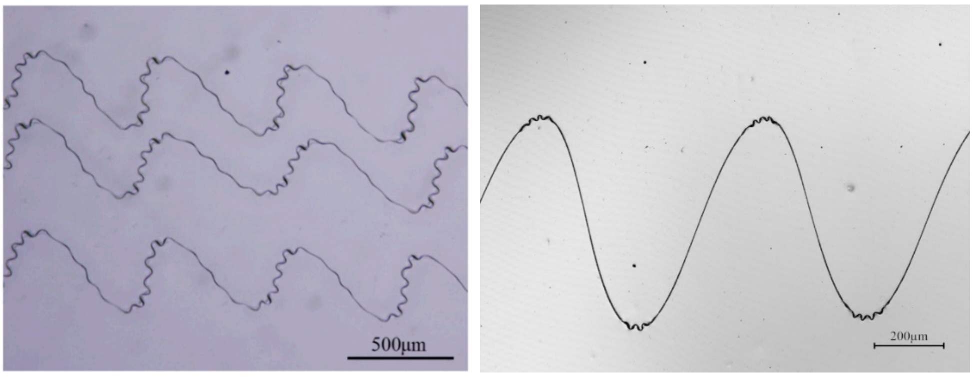

Figure S2. Self-organized buckling of serpentine fibers on a pre-strained elastomer with different tensile strains in two directions.

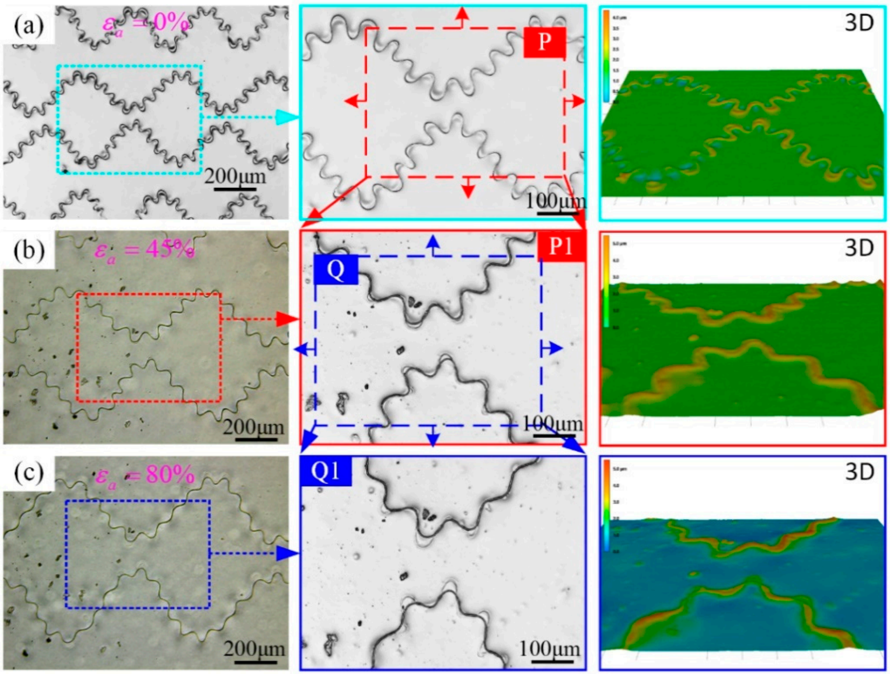

Figure S3. The 2D and 3D LSCM images of the shape geometry of self-similar fiber with applied strain of (**a**) 0%, (**b**) 45%, and (**c**) 80%. The left column is optical image of practical deformation, the middle one is magnification of the left one, and the right one is corresponding 3D image. The second-level waves become large and sparse, and gradually evolved into the initial serpentine structure with the increase of the applied strain. The 3D views show the fibers always deform in-plane, without out-of-plane wrinkling.

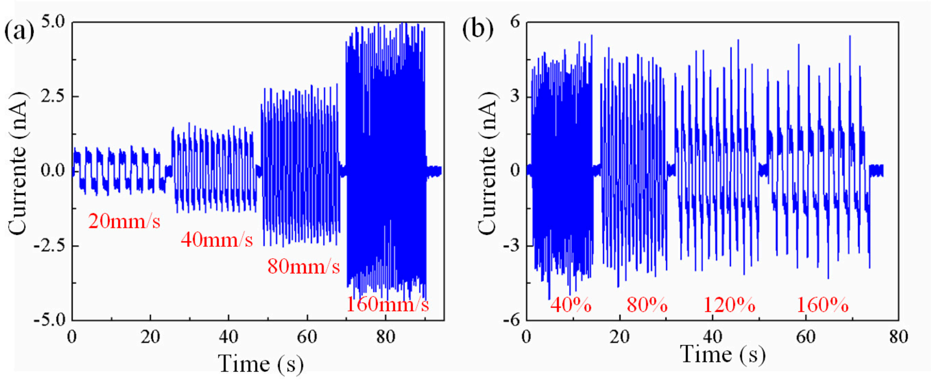

Figure S4. (**a**) The output current of 120% stretching in biaxial direction under 20 mm/s, 40 mm/s, 80 mm/s, and 160 mm/s. (**b**) The output current of 40%, 80%, 120%, and 160% stretching in the biaxial direction, under the same velocity of 40 mm/s.

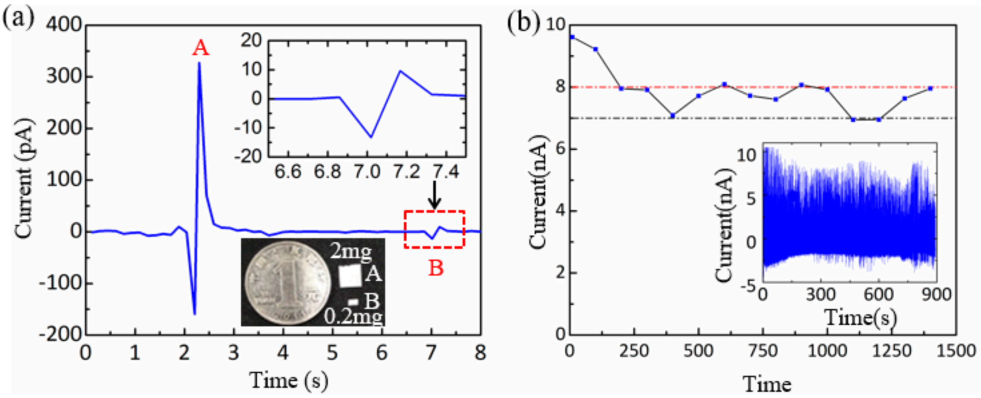

Figure S5. (**a**) The nanogenerator can detect the impact of a lightweight piece of paper. (**b**) Reliability test of the nanogenerator under an applied strain of 150%.

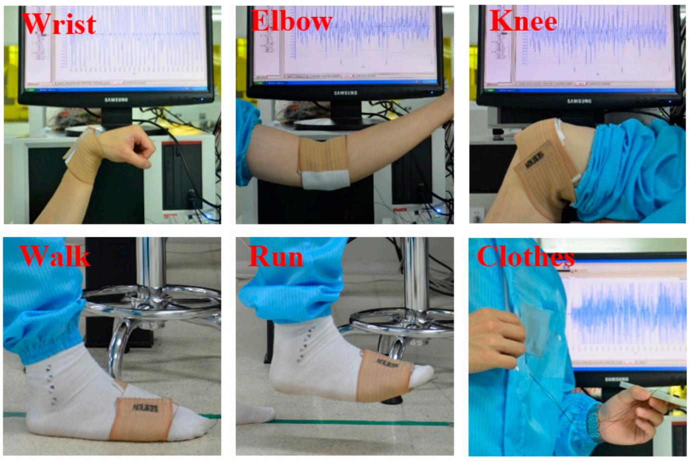

Figure S6. The nanogenerators attached to various body parts with different motions.
